# The Potential Therapeutic Role of *Lactobacillaceae rhamnosus* for Treatment of Inflammatory Bowel Disease

**DOI:** 10.3390/foods12040692

**Published:** 2023-02-05

**Authors:** Hang Guo, Leilei Yu, Fengwei Tian, Wei Chen, Qixiao Zhai

**Affiliations:** 1 State Key Laboratory of Food Science and Technology, Jiangnan University, Wuxi 214122, China; 2 School of Food Science and Technology, Jiangnan University, Wuxi 214122, China; 3 National Engineering Research Center for Functional Food, Jiangnan University, Wuxi 214122, China

**Keywords:** *Lactobacillaceae rhamnosus*, inflammatory bowel disease, gut microbiota, intestinal barrier, immune response, adhesion

## Abstract

Inflammatory bowel disease (IBD) is a heterogeneous group of diseases associated with chronic inflammation of the intestinal tract, and is highly prevalent worldwide. Although its origin is not yet fully understood, new evidence emphasizes that environmental factors, especially dietary factors and intestinal microbiota disorders are key triggers of IBD. Probiotics, such as *Lactobacillaceae* spp., play an essential role in human health as they exert beneficial effects on the composition of the human gastrointestinal microbial community and immune system. Probiotic-based therapies have been shown to be effective in alleviating IBD. Among these, *Lactobacillaceae rhamnosus* is one of the most widely used strains. *L. rhamnosus* is widely present in the intestines of healthy individuals; it regulates the intestinal immune system and reduces inflammation through a variety of mechanisms. The purpose of this study was to identify scientific evidence related to *L. rhamnosus* and IBD, review and summarize the results, and discuss the possible mechanisms of action as a starting point for future research on IBD treatment.

## 1. Introduction

Inflammatory bowel disease (IBD) is a chronic and relapsing intestinal inflammation that manifests in two main clinical phenotypes: ulcerative colitis (UC) and Crohn’s disease (CD). UC mainly involves confluent inflammation of the colonic mucosa, whereas CD usually has a transmural, lamellar character. CD can involve any portion of the gastrointestinal tract, ranging from the mouth to the anus [1]. This disease is more common in Western countries, with an incidence of >0.3% [2]. However, recent studies have shown that the prevalence of IBD has increased in countries with improved socioeconomic status [3]. This phenomenon suggests that lifestyle, dietary and environmental changes may contribute to the upward trend of these diseases, especially in genetically susceptible individuals [4]. The etiology of IBD is unknown, but genetic and other factors (e.g., microbiota and diet) seem to lead to immune disorders, including altered immune responses to the microbiota, disruption of the gut barrier function and ultimately, chronic inflammation [5,6]. The intestinal epithelium separates the intestinal lumen from the immune cells. Microorganisms and antigens affect immune cells by contact with or crossing the intestinal epithelial barrier to induce an immune response [7].

The treatment of IBD includes anti-inflammatory, immunomodulatory and immunosuppressive drugs, in addition to bio-therapy targeting inflammatory cytokines, such as tumor necrosis factor (TNF) or blocking immune cell homing [8]. However, these therapies have significant side effects and high treatment costs [9,10]. A 2018 meta-analysis of 27 population-based randomized controlled trials found that probiotic supplementation was beneficial for both adult IBD and pediatric IBD relative to traditional treatments. The results of seven trials on CD suggest that probiotics may have a significant effect on CD in general, and in particular in post-surgical CD. In UC, probiotic supplementation remains effective for symptom relief. One of them, VSL#3—a mixture of probiotics and prebiotics, has a very significant effect on UC patients [11,12,13,14]. In addition, great progress has been made in understanding the mechanism of the effects of different probiotic strains and their relationship with IBD [15]. Probiotics have been reported to alleviate colitis symptoms by regulating gut microbiota [16], enhancing the mucosal barrier effect [17], inhibiting the expression of inflammatory cytokines [16] and regulating the imbalanced immune response of the immune system [18].

**Figure 1:**
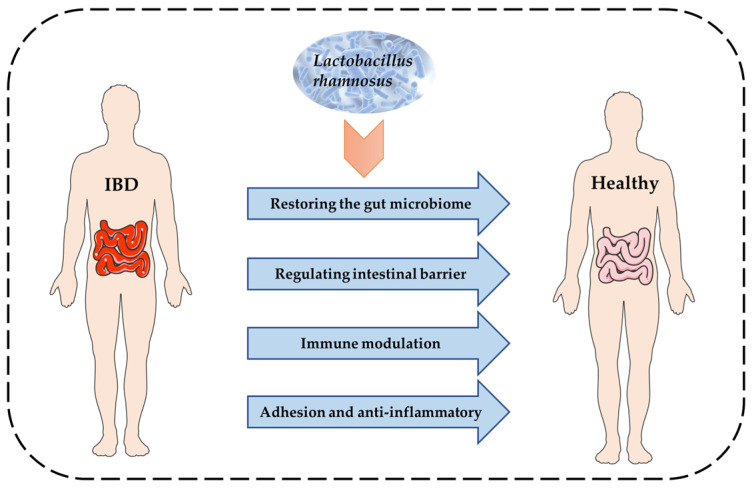
Four main possible mechanisms of IBD alleviation by *L. rhamnosus*.

*L. rhamnosus* is a gram-positive bacterium present in many types of fermented foods and is capable of surviving in a variety of ecological niches, including the intestine and vagina, [19] with a wide range of probiotic properties [20,21]. It has been reported that *L. rhamnosus* can attach to and colonize the surface of the intestinal mucus barrier, thus preventing pathogenic microorganisms from entering the digestive tract and then crossing the intestinal barrier to invade the body [22]. *L. rhamnosus* can also significantly decrease the abundance of pathogens, such as *Escherichia coli* and *Staphylococcus* by metabolizing and producing antibacterial substances in the gut [23]. As shown in Figure 1, several possible mechanisms of action of *L. rhamnosus* may contribute to its beneficial impact on IBD. First, *L. rhamnosus* has been reported to modulate the immune response of intestine-associated lymph-like and epithelial cells via bacterial products [24] and cell wall components [25]. Second, *L. rhamnosus* may affect the intestinal barrier function by the secretion of mucin [26], regulation of epithelial cell apoptosis [27] or expression of tight-junction proteins [28]. Furthermore, *L. rhamnosus* may influence the composition of gut microbiota through competition for nutrients and mucosal adhesion [29]. In this paper, we summarize recent studies of *L. rhamnosus* in different IBD models, as shown in Table 1 and Table 2.

## 2. Molecular Mechanism of *L. rhamnosus* to Alleviate IBD

### 2.1. IBD and Gut Microbiota

#### 2.1.1. Relationship between IBD and Gut Microbiota

Disturbances in the composition of gut microbiota may lead to a dysregulated immune response and altered gut barrier function, resulting in chronic inflammation of the intestine. There is sufficient evidence that gut microbiota is associated with the development of IBD. Studies have shown that fecal shunting ameliorates intestinal inflammation in CD [45]. Additionally, antibiotics are somewhat effective in the treatment of IBD [46]. Under sterile conditions, the disease either did not develop or was significantly milder, suggesting that microorganisms are critical for the progression of intestinal inflammation in colitis [47]. In addition, the composition and diversity of the gut microbiota are altered in patients with active IBD, compared to those in healthy individuals [48]. Moreover, many of the reported IBD susceptibility genes are related to microbial identification and disposal [49].

#### 2.1.2. Alleviating Symptoms in Animal Models by Regulating Gut Microbiota

The current theory of the inflammation-driven dysregulation mechanism suggests that increasing the level of lumen oxygen from the leaking intestine will induce the overgrowth of facultative anaerobes and severely inhibit anaerobic bacteria [50], patients with IBD show a reduction in biodiversity, decreased stability and expansion of proteobacteria. The loss of microbiota diversity may result in a reduction or even the loss of key functions essential for maintaining the integrity of the gut barrier and regulating the host immune system, which may lead to increased inflammation and an immune response [51,52]. To date, no specific pathogenic bacteria have been identified in association with IBD.

Moreover, probiotics, as a strategy to treat intestinal disorders, that reduce the pathogenic bacteria [53] and fungi [54] have also been studied in colitis. In a recent study, *L. rhamnosus* LDTM 7511 attenuated the release of inflammatory cytokines, in addition, it induced a shift in the gut microbiota from a dysregulated state, showing an opposite pattern to that of the DSS group in the abundance of bacterial taxa associated with DSS colitis [33]. *L. rhamnosus* SHA113 increased the abundance of SCFA-producing genera (e.g., *Bifidobacterium*, *Akkermansia* and *Olsenella*) and decreased the abundance of harmful bacteria in the intestine of UC mouse models. It also increased the number of genera positively correlated with mucin expression (*Prevotella*, *Enterorhabdus* and *Parvibacter*) and restored the mucus barrier, resulting in the alleviation of disease symptoms [35]. Furthermore, a recent study found that the combination of *L. rhamnosus* 1.0320 with inulin increased the abundance and diversity of the gut microbiota and increased the content of beneficial bacteria (e.g., *Akkermansia muciniphila*) more than *L. rhamnosus* 1.0320 alone. *Akkermansia muciniphila* was present in the combination group, suggesting that it has anti-inflammatory effects in mice. [31]. In addition, exopolysaccharides from *L. rhamnosus* have been reported to modulate the gut microbiota in IBD. In a recent report, researchers found that exopolysaccharides produced by *L. rhamnosus* ZFM231, significantly attenuated DSS-induced IBD symptoms, which is attributable to its ability to regulate the structural composition of the gut microbiota and maintain gut homeostasis by promoting the abundance of anti-inflammatory bacteria [55]. In conclusion, these studies suggest that *L. rhamnosus* can promote the restoration of the microbiota structure and function by modulating the abundance of specific bacteria in the gut in IBD, leading to improvements in the disease.

### 2.2. IBD and the Intestinal Barrier

#### 2.2.1. Relationship between IBD and Abnormal Intestinal Barrier Function

Intestinal epithelial cells (IECs) form a protective barrier against luminal contents and the external environment via tight intercellular junctions (TJs). This protective barrier prevents the permeation of pro-inflammatory mediators from the luminal environment into the mucosal tissue, and ultimately into the circulatory system [56,57]. There is growing evidence that abnormal epithelial barrier function plays a key part in the pathophysiology of IBD [58]. Clinical studies have shown that intestinal barrier dysfunction precedes IBD, and that intestinal permeability, serum antibacterial antibodies and specific proteins can be used as prognostic indicators of disease and precede clinical diagnosis by several years [59,60]. In normal intestinal tissues, calmodulin, which belongs to adherens junctions, forms dimers with calmodulin on adjacent cells and tight junction proteins, in the upper part of adherens junctions, enhancing cell-to-cell binding. In the inflamed intestine, the expression of these connexins is generally downregulated, with the exception of Claudin-2 [61].

#### 2.2.2. Ability to Restore Intestinal Barrier Function in Animal and Cellular Models

One of the key protective effects of probiotics on intestinal tissues is the strengthening of epithelial tight junctions and ensuing maintenance of intestinal barrier function. It has been reported that probiotics enhance barrier function not only by inducing the synthesis and assembly of tight junction proteins [62], but also by preventing harmful factors from destroying tight junctions [63]. In addition, probiotics release bioactive factors that trigger the activation of multiple cellular signaling pathways, thereby enhancing tight junctions and barrier function [64].

*L. rhamnosus* GG (LGG) can prevent oxidative stress-induced destruction of tight junctions and barrier functions in Caco-2 cell monolayers [65]. In a recent study, the anti-inflammatory properties of *L. rhamnosus* CNCM I3690 were confirmed using an in vitro model of TNF-α-stimulated Caco-2 monolayer cells, and its protective effect on epithelial function was noted. Subsequently, the authors tested the protective effect of *L. rhamnosus* CNCM I-3690 in a mouse model with increased colon permeability and found that *L. rhamnosus* CNCM I-3690 was able to restore partial function of the gut barrier and increase the levels of tight junction proteins occludin and E-cadherin [28]. As described in another study, *L. rhamnosus* SHA113 increased the expression levels of mucin in colon tissue, while significantly increasing the expression of ZO-1, thereby restoring intestinal barrier function in mice [35]. In addition, LGG-derived soluble proteins (p40 and p75) also defend the intestinal barrier against hydrogen peroxide-induced injury in an extracellular signal-regulated kinase (ERK)1/2 mitogen-activated protein kinase (MAPK)-dependent manner by enhancing membrane translocation of tight junction complex proteins, including PKCβ1, ZO-1, and occlusion [65]. Oral administration of live and heat-inactivated *L. rhamnosus* OLL2838 restored the barrier function and prevented the DSS-induced colitis-induced mucosal permeability. This may be due, at least partially, to the increased expression of myosin light chain kinase and ZO-1 in the intestinal epithelial cells [36].

### 2.3. IBD and the Immune Function Modulation

#### 2.3.1. Relationship between IBD and the Abnormal Immune Function

Normal innate and adaptive immune regulation maintains a healthy state by balancing changes in the host physiology caused by antigens in the diet or by bacteria, viruses, fungi and parasites in the gut. If these evolved adaptive mechanisms fail or diminish owing to changes in environmental factors, such as host lifestyle, chronic intestinal inflammation can occur [66]. Thus, maintaining a dynamic balance between necessary and excessive immune defenses is an effective therapy for IBD. Innate immune responses are elicited by host pattern recognition receptors (PRRS) on leukocytes that recognize bacterial pathogen-associated molecular patterns (PAMPs). Their pro-inflammatory responses are thought to underlie the pathogenesis of IBD [67]. Activation of adaptive immunity is due to the innate immune deficiency of an organism. Patients in infancy have abrogated IL-10 signaling and exhibit a phenotype similar to that of IBD. In these patients, macrophage responsiveness to IL-10 is lost because of IL-10 receptor defects or defects in IL-10 production by monocytes/macrophages and regulatory T cells [68].

Maintenance of pathogen-host homeostasis through Toll-like receptors (TLRs) is an important way that probiotics exert their efficacy [69]. Many studies have demonstrated that TLR signaling directly impacts the function and proliferation of Treg cells [28]. In addition to TLRs, researchers have found that the pro-inflammatory cytokine environment and specific transcription factors are essential for the regulation of Th17/Treg homeostasis [70,71]. Th17 cells are a subset of Th cells that produce IL-17 and are implicated in the development and progression of many inflammatory responses and autoimmune diseases [72]. Many Foxp3^+^ T cells in the intestinal tract produce high levels of RoRct and IL-17. Over 25% of IL-17^+^ T cells produce Foxp3 at a certain stage of their development [73]. For example, the immunomodulatory effect of LGG on pathogenic *Porphyromonas gingivalis* is mediated by the maintenance of Th17 and Treg homeostasis. Through activation of the TLR4-mediated signaling pathway, *Porphyromonas gingivalis* caused an increase in Th17 cells and pro-inflammatory factors. Immediately upon balance disruption, LGG increased the proportion of Treg cells through the TLR2 signaling pathway and decreased the proportion of Th17 cells in the CD4^+^ T cell system to maintain a steady state [74].

#### 2.3.2. Improving Immune Disorders Caused by Colitis in Animal Models

Oral administration of *L. rhamnosus* HDB1258 can enhance the immune response by activating the innate immunity of the host, including macrophage phagocytosis and NK cell cytotoxicity, and by regulating IL-10 and TNF-α in the intestinal microbiota and immune cells to inhibit systemic inflammation in inflammatory hosts [75]. The strong antioxidant activity of *L. rhamnosus* inhibits reactive oxygen species (ROS) production and phagocytosis of neutrophils, as well as protects cells from cytotoxic damage [76]. ROS can regulate inflammatory signaling by instantly oxidizing catalytic cysteine residues in key regulatory enzymes [77]. Lin et al. [78] reported that LGG blocked the activation of the pro-inflammatory transcription factor NF-κB in the distal small intestine of immature mice by inducing the release of ROS in epithelial cells and blocking Cul1-deacetylation required for the activation of the ubiquitin ligase complex. Another study revealed that pilin subunit (SpaC), which mediates the adhesion of *L. rhamnosus* [79], contributes to LGG-induced ROS production in epithelial cells [80]. A study reported that *L. rhamnosus* 1.0320 treatment resulted in the decreased expression of IL-1β, IL-, and TNF-α, and increased IL-10 levels [31]. All of these cytokines have been reported to be associated with the pathogenesis of IBD [81]. In addition, Rodrigues et al. [32] observed that *L. rhamnosus* EM1107 intake significantly reduced the levels of pro-inflammatory cytokines in rats with colitis, thus contributing to the improvement of inflammation.

### 2.4. IBD and Adhesion Anti-Inflammatory

#### 2.4.1. IBD and Inflammation Caused by Adherent Pathogenic Bacteria

In previous studies, adherent invasive *Escherichia coli* has been repeatedly reported to be associated with the pathogenesis of IBD, particularly CD. Several studies have isolated adherent *E. coli* from the ileal mucosa of patients with CD; However, no virulence factors have been detected in the genes of typical pathogenic species, which is one of the characteristics of their high prevalence [82,83]. The common characteristic of these strains is the adhesion and invasion of intestinal epithelial cells by specific adhesion factors and the induction of increased IL-8 production by epithelial cells, resulting in an inflammatory response in intestinal tissues. This has been reported for enterohemorrhagic *E. coli* strains to occur via the adhesin AAF or for diffusely adhering *E. coli* strains via the Afa/Dr adhesions [84,85,86,87].

In addition, a recent study showed that inflammation caused by enterohemorrhagic *E. coli* disrupts the balance between pro- and anti-inflammatory proteins [82]. Extracellular factors (including flagella), elicit an inflammatory response that ultimately leads to tissue inflammation [88].

Probiotics that colonize the gastrointestinal tract competitively inhibit the adhesion of pathogens to the intestinal epithelium by occupying ecological niches. Studies have shown that the adhesion between probiotics and intestinal epithelial cells is mediated by lectin-like and cell surface protein components [89]. Therefore, probiotics or combinations of probiotics for specific disease types should be selected based on their capacity to inhibit or replace specific pathogens [90].

#### 2.4.2. LGG Exerts Adhesion and Anti-Inflammatory Effects in Animals and In Vitro Models

Recent evidence showed that LGG adheres to the colonic mucosa and exerts local anti-inflammatory effects both in in vitro and in vivo experimental models. In the in vitro organ culture models, LGG exhibited consistent adhesion and anti-inflammatory effects. Furthermore, LGG colonization was confirmed in the human colon one week after consumption. In addition, a reasonable increase in the dose can increase the adhesion and effectiveness of LGG [91]. Studies have shown that the pilus of LGG was capable of producing a mucus-binding protein, which enhances its adhesion properties [92]. Furthermore, SpaC helps LGG induce ROS production in epithelial cells and enables the LGG strain to stimulate intracellular extracellular signal-regulated kinase/mitogen-activated protein kinase (ERK)/MAPK signaling in enterocytes [81]. p40 is an LGG-secreted protein that protects intestinal epithelial cells from inflammation. Another study found that adding a colonic epithelial cell-derived component to the growth medium of LGG significantly promoted the synthesis and secretion of the p40 protein and enhanced the protective response of LGG-stimulated intestinal epithelial cells [24]. In addition, another study compared whether this treatment had an effect on relieving inflammation by inactivating the pili operon of *L. rhamnosus* CNCM I-3690: spaFED. It was found that colonic cytokine levels, colonic permeability, goblet cell (GC) populations and lymphocyte populations in mice with induced hypo-inflammation treated with the Δ*spaF* mutant remained significantly different from those in controls treated with wild-type *L. rhamnosus* CNCM I-3690 [38]. This evidence demonstrates that the ciliated structure of *L. rhamnosus* underlies its interaction with other bacteria in the intestine and its importance for the protection of the intestinal environment.

## 3. Safety Issues in the Application of *L rhamnosus*

Although probiotics have been used in clinical practice for decades, some safety issues have arisen due to the exponential growth in their use and ease of availability in recent years. Several studies have reported safety concerns associated with the use of *L. rhamnosus*. Recently, one study reported that LGG exacerbated intestinal ulcers in an anti-inflammatory, pain-induced enteropathy model [93]. Some studies have reported LGG overexpression-induced bacteremia, although these studies examined specific populations, such as young children or patients with metabolic diseases [94]. To better understand the potential and rare adverse events associated with *L. rhamnosus* consumption, it is essential to focus on its strain specificity [95]. In fact, the phenotypic differences observed among different strains of *L. rhamnosus* compared to the already commercialized LGG may be related to the shift from the commensal to opportunistic type of *L. rhamnosus* isolated from bacteremia [96]. However, no studies have assessed the shift from symbiotic to opportunistic species. Therefore, an in-depth study of published clinical records and case reports remains the best way to avoid the adverse reactions associated with the use of *L. rhamnosus*. In addition, there is an urgent need for regulatory agencies to establish a consistent regulatory framework for probiotic products, with unified management of production specifications, ingredient labeling, efficacy promotion and risk identification.

## 4. Conclusions and Outlook

**Figure 2:**
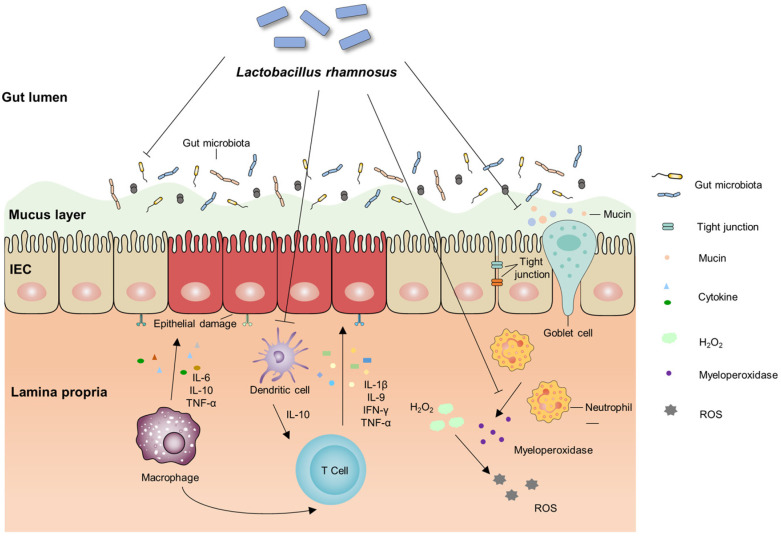
Protective mechanism of *L. rhamnosus* against intestinal inflammation. *L. rhamnosus* protects the mucus barrier of the intestine by stimulating the production of mucins from globlet cells, and also reduces intestinal inflammation by inhibiting the production of pro-inflammatory cytokines and ROS. In addition, *L. rhamnosus* can directly regulate gut microbiota and promote the proliferation of beneficial bacteria, thus restoring microbiota function.

An increasing number of studies have shown that *L. rhamnosus* can exert a palliative effect on IBD through multiple mechanisms (Figure 2). Despite the anti-inflammatory effects of *L. rhamnosus* in in vitro and in vivo animal models, clinical trial evidence to date does not support its large-scale application, and further systematic and rigorous large-scale clinical cohort studies are needed. In these large clinical trials, the feasibility, effectiveness, adverse events and long-term safety issues of the interventions need to be evaluated to ensure responsible use. These large clinical trials need to be informed by the rigorous testing and approval processes in place for other human interventions, ensuring a uniform and unbiased approach to experimentation and regulation. Well-designed clinical trials that investigate specific mechanisms of action of probiotics in the pathogenesis of IBD will provide new therapeutic options in the future. We expect that future studies will determine the optimal dose and correct combination of various probiotics that will match the molecular and cellular pathogenesis of gastrointestinal diseases. These studies may help improve the efficacy of probiotic approaches. Hopefully, this will lead to the development of a new generation of probiotics with scientific evidence of their health benefits.

## Figures and Tables

**Table 1:** Publications showing the results of using probiotics for IBD in animal models.

Ref.	Numbers, Model, Age	Moulding Method	Probiotic Strains	CFU/Dose, Duration	Effects
[30]	25, Dark Agouti rats, 30 d	TNBS	*L. rhamnosus* 64	3 × 10^6^ CFU, daily, 27 d	damage score↓, immune cell infiltration↓, cytokine↓, MPO activity↓.
[31]	90, BALB/c mice, 7 w	DSS	*L. rhamnosus* 1.0320	2 × 10^8^ CFU, daily, 28 d	the DAI score↓, hemoglobin content↑, MPO activity↓, IL-1β↓, IL-6↓, TNF-α↓ and IL-10↑.
[32]	48, Wistar rats, 10 w	Acid	*L. rhamnosus* EM1107	10^9^ CFU, daily, 17 d	TNF-α↓, myeloperoxidase↓, IL-1β↓and oxidative stress↑. IL-17↓, NF-κB p65↓, MMP-2↓, MMP-9↓, and iNOS↓, SOCs-1↑, ZO-1↑ and mucin-2↑.
[33]	32, C57BL/6J mice, 6 w	DSS	*L. rhamnosus* LDTM 7511	10^9^ CFU, daily, 14 d	colon length↑, spleen weight↓, Lcn-2↓, MPO↓, CRP↑, relatively intact colonic architecture, Chao1 index↑, Shannon index↑.
[34]	40, C57BL/6 mice, 8 w	DSS	*L. rhamnosus* L34	1 × 10^7^ CFU, once every 3 d, 14 d	the gut local inflammation↓, gut-leakage severity↓, fecal dysbiosis↓ and systemic inflammation↓.
[35]	40, C57BL/6Cnc mice, 8 w	DSS	*L. rhamnosus* SHA113	10^9^ CFU, 9 d	SCFA-producing genera↑, UC-related genera↓.
[36]	16, BALB/c mice, 6 w	DSS	*L. rhamnosus* OLL2838	10^7^ CFU, 3 d	Body weight↑, and colon length↑, expression of zonula occludens-1 and myosin light-chain kinase↑
[37]	40, C57BL/6J mice, 5 w	DSS	*L. rhamnosus* Hao9	10^9^ CFU, 7 d	DAI↓, colon length↑, alleviated colonic pathological variations, histological scores↓, TNF-α, IL-6, and IL-1β ↓, IL-10↑.
[38]	50, C57BL/6 mice	DNBS	*L. rhamnosus* I-3690	5 × 10^9^ CFU, 10 d	macroscopic scores↓, cytokine levels↓, colon and ileum MPO activities↓

Damage score: Crude morphological scoring of the isolated colonic tissue [30]. Disease activity index (DAI) score: A composite score combining three conditions: percent weight loss, stool consistency, and stool bleeding [31]. DSS: dextran sulfate sodium salt; TNBS: trinitro-benzene-sulfonic acid; MPO: myeloperoxidase; IL-: interleukin-; TNF-α: tumor necrosis factor-; MMP-: matrix metalloproteinase-; ZO-1: zonula occludens-1; CRP: C-reactive protein; Lcn-2: lipocalin– 2/NGAL;.

**Table 2:** Publications showing the results of using probiotics for IBD in human models.

Ref.	Numbers of Patients	Disease	Probiotic Strains	CFU/Dose, Duration	Effects
[39]	187 adults	UC	LGG	18 × 10^9^ CFU, 12 m	LGG treatment is more efficient than standard treatment in extending relapse-free time.
[40]	4 children	CD	LGG	10^10^ CFU, twice a day, 6 m	The median activity index of children with CD at 4 weeks was 73% lower than at baseline.
[41]	11 adults	CD	LGG	2 × 10^9^ CFU, daily, 6 m	The median time to relapse was on average 4 weeks longer in the LGG group than in the placebo group.
[42]	14 children	CD	LGG	10^10^ CFU, twice a day, 10 d	Oral administration of LGG increased the intestinal IgA immune response, thus promoting the intestinal immune barrier.
[43]	37 adults	CD	LGG	6 × 10^9^ CFU, twice a day, 52 w	LGG appears to neither prevent one-year endoscopic recurrence nor reduce the severity of recurrent disease.
[44]	117 adults	CD&UC	LGG	1.4 × 10^10^ CFU, daily, 52 w	Patients taking daily LGG had a lower frequency of first postoperative inflammatory bowel disease complications.
